# Genetic regulatory subnetworks and key regulating genes in rat hippocampus perturbed by prenatal malnutrition: implications for major brain disorders

**DOI:** 10.18632/aging.103150

**Published:** 2020-05-11

**Authors:** Jiaying Chen, Xinzhi Zhao, Li Cui, Guang He, Xinhui Wang, Fudi Wang, Shiwei Duan, Lin He, Qiang Li, Xiaodan Yu, Fuquan Zhang, Mingqing Xu

**Affiliations:** 1 Bio-X Institutes, Key Laboratory for the Genetics of Developmental and Neuropsychiatric Disorders (Ministry of Education), Shanghai Jiao Tong University, Shanghai 200030, China; 2 Shanghai Key Laboratory of Psychotic Disorders, Shanghai Mental Health Center, Shanghai Jiao Tong University School of Medicine, Shanghai 200030, China; 3 Center for Biomedical Informatics, Harvard Medical School, Boston, MA 02115, USA; 4 International Peace Maternity and Child Health Hospital of China Affiliated to Shanghai Jiao Tong University, Shanghai 200030, China; 5 Shanghai Key Laboratory of Veterinary Biotechnology, School of Agriculture and Biology, Shanghai Jiao Tong University, Shanghai 200240, China; 6 School of Public Health, The First Affiliated Hospital, Institute of Translational Medicine, Zhejiang University School of Medicine, Hangzhou 310058, China; 7 Medical Genetics Center, School of Medicine, Ningbo University, Ningbo 315000, China; 8 Translational Medical Center for Development and Disease, Institute of Pediatrics, Shanghai Key Laboratory of Birth Defect, Children's Hospital of Fudan University, Shanghai 201102, China; 9 Department of Developmental and Behavioral Pediatrics, Pediatric Translational Medicine Institute, Shanghai Children′s Medical Center, Shanghai Jiao Tong University School of Medicine, Shanghai 200127, China; 10 Department of Psychiatry, The Affiliated Brain Hospital of Nanjing Medical University, Nanjing 210029, China

**Keywords:** prenatal, famine, malnutrition, rat, transcriptome

## Abstract

Objective: Many population studies have shown that maternal prenatal nutrition deficiency may increase the risk of neurodevelopmental disorders in their offspring, but its potential transcriptomic effects on brain development are not clear. We aimed to investigate the transcriptional regulatory interactions between genes in particular pathways responding to the prenatal nutritional deficiency and to explore their effects on neurodevelopment and related disorders.

Results: We identified three modules in rat hippocampus responding to maternal prenatal nutritional deficiency and found 15 key genes (*Hmgn1, Ssbp1, LOC684988, Rpl23, Gga1, Rhobtb2, Dhcr24, Atg9a, Dlgap3, Grm5, Scn2b, Furin, Sh3kbp1, Ubqln1, and Unc13a*) related to the rat hippocampus developmental dysregulation, of which *Hmgn1, Rhobtb2* and *Unc13a* related to autism, and *Dlgap3, Grm5, Furin* and *Ubqln1* are related to Alzheimer’s disease, and schizophrenia. Transcriptional alterations of the hub genes were confirmed except for *Atg9a*. Additionally, through modeling miRNA–mRNA-transcription factor interactions for the hub genes, we confirmed a transcription factor, Cebpa, is essential to regulate the expression of *Rhobtb2*. We did not find singificent singals in the prefrontal cortex responding to maternal prenatal nutritional deficiency.

Conclusion: These findings demonstrated that these genes with the three modules in rat hippocampus involved in synaptic development, neuronal projection, cognitive function, and learning function are significantly enriched hippocampal CA1 pyramidal neurons and suggest that three genetic regulatory subnetworks and thirteen key regulating genes in rat hippocampus perturbed by a prenatal nutrition deficiency. These genes and related subnetworks may be prenatally involved in the etiologies of major brain disorders, including Alzheimer’s disease, autism, and schizophrenia.

Methods: We compared the transcriptomic differences in the hippocampus and prefrontal cortex between 10 rats with prenatal nutritional deficiency and 10 rats with prenatal normal chow feeding by differential analysis and co-expression network analysis. A network-driven integrative analysis with microRNAs and transcription factors was performed to define significant modules and hub genes responding to prenatal nutritional deficiency. Meanwhile, the module preservation test was conducted between the hippocampus and prefrontal cortex. Expression levels of the hub genes were further validated with a quantitative real-time polymerase chain reaction based on additional 40 pairs of rats.

## INTRODUCTION

A multitude of epidemiological studies, which were based on the Dutch Hunger Winter Famine [1] and the Chines Famine [2] cohorts, demonstrated that maternal exposure to nutrition deficiency during critical stages of pregnancy significantly increases the risks of schizophrenia, bipolar disorder in the offspring. Different maternal nutritional deficiencies during pregnancy probably lead to different types of neurodevelopmental disorders in the offspring. Lack of several common nutrients includes folate, essential fatty acids, retinoids, vitamin D, iron and protein-calorie malnutrition (PCM) [3] before delivery can lead to neural tube defects in offspring [4], decreased IQ in childhood, central nervous system malformation, neuronal cell growth and differentiation disorder, nerve junction and myelin sheath defects, as well as neurotransmitters, cells, electrophysiology and behavioral disorders [3, 5], respectively.

Animal-based research showed that maternal prenatal nutrition deficiency is closely related to the neurodevelopment of offspring [6, 7]. Protein malnutrition has been extensively studied as an important research direction of maternal malnutrition during pregnancy. We have established a prenatal malnutrition (famine) rat model, named RLP50, which was induced by prenatal exposure to a diet restricted to 50% of a low-protein (6%) [8]. Using gene expression and DNA methylation modifications profiling strategies, we have observed significantly different patterns of gene expression and trace elements in pregnant rats of the RLP50 group. This broadens our understanding of the complex biochemical perturbations that prenatal exposure to famine can induce, and these perturbations may eventually lead to impairment of fetal neurodevelopment [8]. However, these studies emphasized only on screening differentially expressed biomarkers rather than determining the connection between them, in which biomarkers with similar expression patterns may be functionally related. Moreover, the regulatory interactions between genes in particular pathways or biological processes responding to the prenatal nutritional deficiency have not been investigated. Additionally, potential novel regulators of transcription and post-transcription dominating prenatal nutritional deficiency-induced gene expression changes, including micro-RNAs, long noncoding RNAs, and transcription factors, have not been investigated how they regulate the transcription-level RNA interactions. Thereby understanding the functional molecular mechanisms regulated by these interactions is essential for gaining biological insights into gene functions. Transcriptomic network analysis enables us to cluster genes by assigning them to known biological functions in which they are involved [9]. Among the transcriptomic network inference algorithms, weighted gene co-expression network analysis (WGCNA) is a relatively new statistical method not only to infer correlation patterns between two genes but also covers neighborhood across expression data through constructing subnetworks named modules [10]. Plenty of evidence suggested the modules as stable units underlying transcriptional regulation networks whose function can remain the same while individual gene expression can be changed or replaced by other genes with similar redundant functions [11].

In this study, we conducted a co-expression network analysis for identifying putative genes and subnetwork responding to the perturbation of prenatal nutrition deficiency in the rat brain hippocampus and prefrontal cortex. A network-driven integrative analysis was performed to find significant modules and a module preservation test was conducted to test the robustness of the significant modules. Further gene ontology and protein-protein interaction analyses were conducted to determine potential hub genes. Through co-expression network analysis, we identified three genetic regulatory subnetworks and fifteen key regulating genes in rat hippocampus followed with independent PCR verification and inference of miRNA–mRNA-transcription factor interactions that dominate prenatal nutritional deficiency-induced gene expression changes, demonstrating functional implications for Alzheimer’s disease, autism, schizophrenia, and other neuropsychiatric disorders.

## RESULTS

### Demographic characteristics of RLP50 and neonatal rats

**Figure 1:**
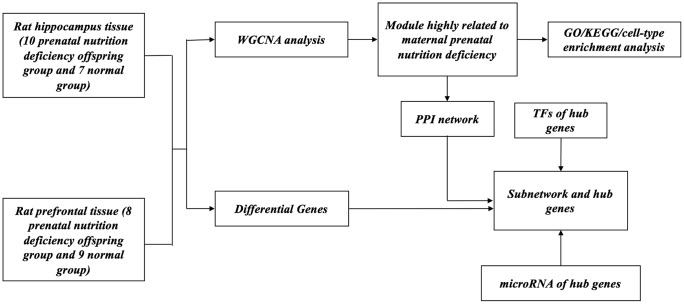
**Flow chart of the analytic procedure.**

Prenatal malnutrition did not significantly alter birth numbers. However, the neonatal birth weights in both the LP and RLP50 groups were lower than those in the control group, with the RLP50 group having the lowest birth weights (*p* < 0.001). Meanwhile, the RLP50 group showed significant maternal weight percent gain compared to the other two groups (*p* < 0.001). As previously reported in our studies [12, 13], we observed a tendency for the LP group to build a smaller nest and a complete disappearance of nest building in the RLP50 group after gestational exposure to simulated famine (p < 0.001), indicating that gestational exposure to malnutrition and the stress of starvation resulted in maternal behavior disruption. Behavior tests of neonatal rats between groups did not find significant differences. The sole purpose of this low protein group was to provide a basis to calculate the appropriate diet for the "RLP50" group, (Figure 1) which was used to simulate the prenatal famine situation, thus only rats in RLP50 and control groups were selected to perform the gene expression profiling analysis.

### DEGs in the hippocampus and prefrontal cortex

After microarray data pre-processing, final data sets included 17 hippocampal tissues of rats (10 prenatal nutrition deficiency offspring groups and 7 normal offspring groups) and 17 prefrontal cortex tissues of rats (8 prenatal nutrition deficiency offspring groups and 9 normal offspring groups). The distribution of gender is not significantly different between the RLP50 group and normal nourished offspring groups (Table 1).

**Table 1:** Samples information included in this study.

**Samples**	**Prenatal Nutritional Status**	**Gender**	**Neonatal Body Weight(g)**
**Prefrontal cortex**			
F3F2P	Prenatal Nutrition Deficiency Offspring	Female	4.65
F3F1P	Prenatal Nutrition Deficiency Offspring	Female	4.83
F6F2P	Prenatal Nutrition Deficiency Offspring	Female	4.91
F2F2P	Prenatal Nutrition Deficiency Offspring	Female	4.71
F7M2P	Prenatal Nutrition Deficiency Offspring	Male	5.11
F5M2P	Prenatal Nutrition Deficiency Offspring	Male	5.01
F6M4P	Prenatal Nutrition Deficiency Offspring	Male	5.08
F7M5P	Prenatal Nutrition Deficiency Offspring	Male	4.67
C2F1P	Control Offspring	Female	6.55
C3F2P	Control Offspring	Female	6.78
C1F3P	Control Offspring	Female	7.12
C4F3P	Control Offspring	Female	7.01
C5M4P	Control Offspring	Male	7.25
C5M2P	Control Offspring	Male	7.56
C3M4P	Control Offspring	Male	7.89
C5M3P	Control Offspring	Male	8.41
C4M3P	Control Offspring	Male	7.08
**Hippocampus**			
F6F2H	Prenatal Nutrition Deficiency Offspring	Female	4.91
F3F2H	Prenatal Nutrition Deficiency Offspring	Female	4.65
F2F2H	Prenatal Nutrition Deficiency Offspring	Female	4.71
F5M2H	Prenatal Nutrition Deficiency Offspring	Male	5.01
F6M4H	Prenatal Nutrition Deficiency Offspring	Male	5.08
F7M4H	Prenatal Nutrition Deficiency Offspring	Male	4.79
F3F3H	Prenatal Nutrition Deficiency Offspring	Female	4.93
F2F1H	Prenatal Nutrition Deficiency Offspring	Female	4.99
F7M3H	Prenatal Nutrition Deficiency Offspring	Male	4.67
F7M2H	Prenatal Nutrition Deficiency Offspring	Male	4.74
C2F2H	Control Offspring	Female	6.89
C4F3H	Control Offspring	Female	7.01
C3M4H	Control Offspring	Male	7.89
C5M3H	Control Offspring	Male	8.41
C4M3H	Control Offspring	Male	6.99
C5F1H	Control Offspring	Female	7.87
C5M1H	Control Offspring	Male	8.23

We analyzed rat hippocampus tissue including 10 prenatal nutrition deficiency offspring individuals and 7 normal offspring individuals, as well as prefrontal cortex including 7 prenatal nutrition deficiency offspring individuals and 9 normal offspring individuals, respectively. We found that 1844 genes were differentially expressed between prenatal nutrition deficiency and control offspring individuals, 209 of which were significantly different after FDR adjustment (FDR-corrected P < 0.05) (Supplementary Figure 2, Supplementary Table 3) in the hippocampus. In the prefrontal cortex, 717 genes were differentially expressed between prenatal nutrition deficiency and control offspring individuals, while, we did not find any differentially expressed genes with significance after FDR adjustment (FDR-corrected *P < 0.05) (Supplementary Table 3), which is consistent with our previous report [12] and suggested the hippocampus is more sensitive to the exposure to prenatal nutrition deficiency. In this current study, we identified a much smaller number of DEGs compared with the previous one, in which 2987 DEGs in the hippocampus and 415 DEGs in the prefrontal cortex were reported, because there was no correction for multiple test performed in that previous study.

### Co-expression modules related to prenatal nutritional status

We conducted a weighted gene co-expression network analysis (WGCNA). Before using the WGCNA R package to construct the network, we used cluster analysis to check the quality of hippocampal and prefrontal cortex samples further and no samples were removed (Supplementary Figure 3). Here, the power of β equaling 15 was selected as the soft threshold for constructing a scale-free network in the hippocampus (Supplementary Figure 4A, 4B), while the power of β equaling 18 is selected for prefrontal cortex (Supplementary Figure 4E, 4F).

**Figure 2:**
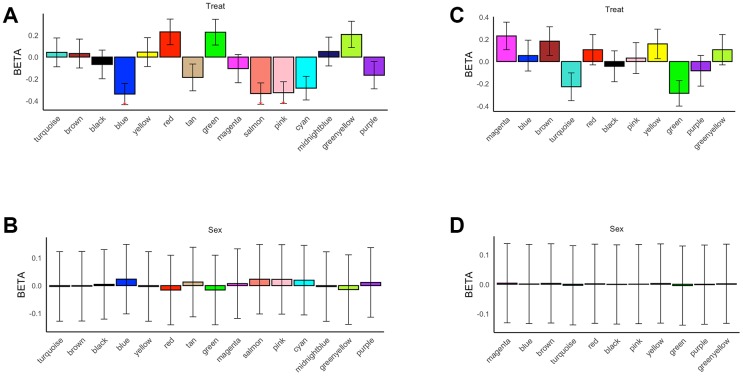
Association test of modules with prenatal nutritional status and gender (FDR-corrected *P < 0.05). (**A**) Module-level differential expression related to prenatal nutritional status in the hippocampus. (**B**) Module-level differential expression related to prenatal nutritional status in the prefrontal cortex. (**C**) Module-level differential expression related to gender status in the hippocampus. (**D**) Module-level differential expression related to gender status in the prefrontal cortex.

In all 17 subjects with hippocampal expression profiling data, 16 modules were identified, 3 of which (blue, pink and salmon modules) showed significant association “Factor (e.g. prenatal nutritional status)” in overall expression patterns (represented by MEs) (Figure 2A), while no module was significantly associated with “Gender” (Figure 2B). In all 16 subjects with prefrontal cortex expression profiling data, 12 modules with co-expressed genes were identified, but no module was significantly correlated with “Factor” (Figure 2C) or “Gender” (Figure 2D).

### Module preservation test

We performed preservation analysis of the expression profiles of the hippocampus and prefrontal cortex and found that there were some weak preserved modules between these two different brain tissues (Supplementary Figure 5), and the findings indicated by medianRank and Zsummary statistics were consistent, which indicated that module size has little effect on preservation analysis. Six hippocampal modules were highly preserved with the prefrontal cortex, including the blue module in the hippocampus, seven modules, including a salmon module in the hippocampus, were moderately preserved in the prefrontal cortex, and two modules, including the pink module in the hippocampus, were weakly preserved with the prefrontal cortex. These findings demonstrated that the gene expression patterns between the hippocampus and prefrontal cortex are different to a large extent, and prenatal malnutrition may easily trigger the changes of gene expression patterns in the hippocampus.

### Enrichment analysis of interesting modules

**Figure 3:**
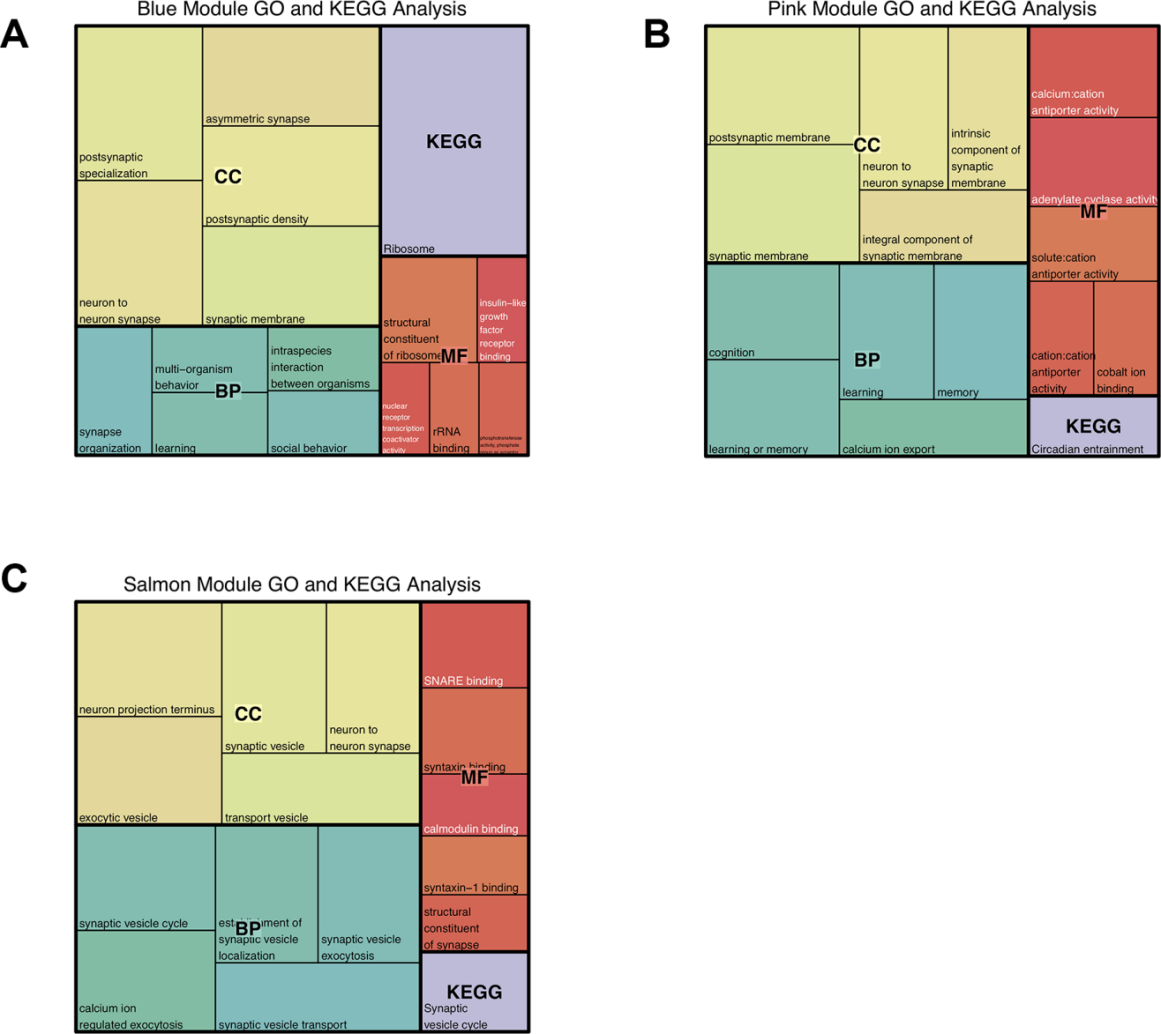
**Gene Ontology and KEGG pathway enrichment analysis of the modules significantly related to prenatal nutrition deficiency in the hippocampus.** (**A**) Top 5 terms significantly enriched in the blue module; (**B**) Top 5 terms significantly enriched in the pink module; (**C**) Top 5 terms significantly enriched in the salmon module.

We conducted gene ontology enrichment analyses of the three modules in the hippocampus significantly related to prenatal nutrition deficiency and found that the module genes related to prenatal nutrition deficiency were involved in neuronal development and function. In the blue module, we found most genes are related to synaptic development, as their cellular component was associated with postsynaptic specialization, neuron to neuron synapse, postsynaptic density, asymmetric synapse, and synaptic membrane. And their genetic functions are related to learning and social behavior (Figure 3A, Supplementary Table 5). In the pink module, we found the cellular components of most genes were related to the synaptic membrane, postsynaptic membrane, and neuron to neuron synapse, etc. Meanwhile, their biological function was involved in learning, memory, and cognition (Figure 3B, Supplementary Table 5). In the salmon module, cellular components of most genes were regarding neuron projection terminus, synaptic vesicle, and neuron projection terminus, etc. Their biological function was major related to synaptic vesicles, such as synaptic vesicle cycle, synaptic vesicle transport, the establishment of synaptic vesicle localization, and synaptic vesicle exocytosis (Figure 3C, Supplementary Table 5). In summary, GO functional enrichment analysis demonstrated that prenatal nutrition deficiency affected the transcriptional expression of neuronal development-related genes.

KEGG enrichment analysis demonstrated that the genes within the blue, pink, and salmon modules were involved in the ribosome, circadian entrainment and synaptic vesicle cycle, respectively (Figure 3, Supplementary Table 6). This further indicated that the salmon module genes are more closely related to synaptic development.

**Figure 4:**
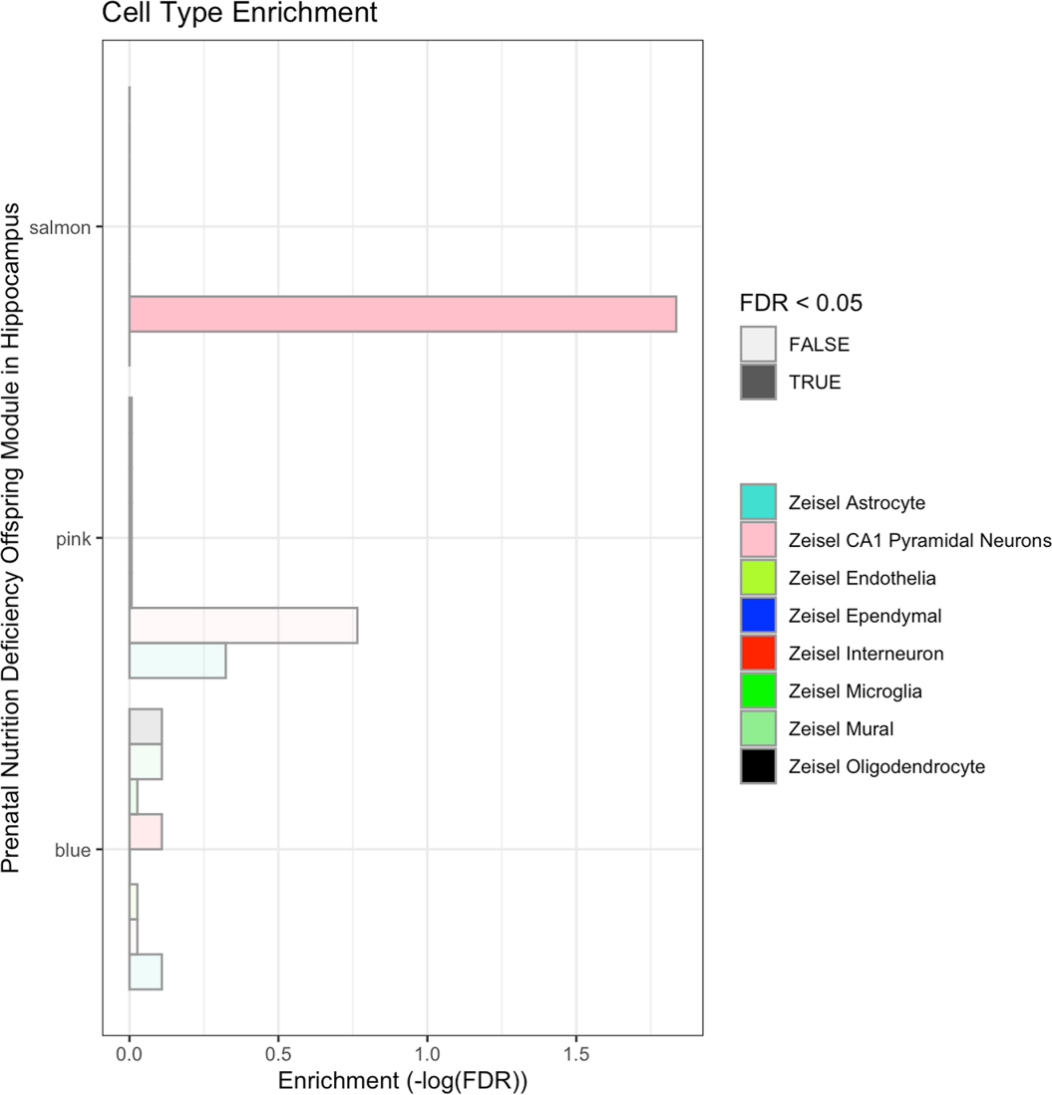
**Cell-type enrichment analysis of the blue, pink and salmon modules significantly responding to prenatal nutrition deficiency in the hippocampus.**

The rat brain hippocampus cell-types enrichment analysis showed that the salmon module was enriched with genes specifically expressed in pyramidal neurons, which are the most common excitable neurons (Figure 4, Supplementary Table 7) sending and receiving nerve impulses within the cerebral cortex, hippocampus, and the amygdala. In mammals, it is thought that pyramidal neurons play a key role in cognitive functions, such as perception, reasoning, remembering, thinking and understanding. We did not find significant enrichment of cell-types in blue and pink modules.

To further evaluate the effects of prenatal nutrition deficiency on the risk of neurodevelopmental diseases, we conducted disease-related gene set enrichment analysis based on the DisGeNET database, in which 24166 human diseases and their related-genes were included. We concentrated on 10 major brain diseases, including attention deficit hyperactivity disorder (ADHD), autism, anoxia, bipolar disorder, Parkinson’s disease, major depressive disorder, Alzheimer’s disease, schizophrenia, and glioma. The results demonstrated that the blue module was significantly overlapped with genes related to Alzheimer's disease, autism, and schizophrenia, while the pink and salmon modules were significantly overlapped with schizophrenia-related genes. These findings suggest that prenatal nutrition deficiency may increase the risk of neurodevelopmental diseases, including Alzheimer's disease, autism, and schizophrenia (Table 2).

**Table 2:** Enrichment analysis of module with genes related to major brain diseases.

**Disease**	**No. Disease Gene**	**Blue Module (795 genes)**			**Pink Module (278 genes)**			**Salmon Module (117 genes)**
		**No. Overlapped Gene**	**P-value**	**Adjusted P Value**	**No. Overlapped Gene**	**P-value**	**Adjusted P Value**	**No. Overlapped Gene**	**P-value**	**Adjusted P Value**
Intellectual Disability	2684	132	6.00E-04	**1.50E-03**	49	1.01E-02	**1.59E-02**	22	3.63E-02	**4.50E-02**
Epilepsy	1643	98	2.93E-06	**2.18E-05**	43	1.04E-05	**3.91E-05**	15	3.68E-02	**4.50E-02**
Autism	1011	66	8.64E-06	**3.70E-05**	28	1.65E-04	**4.96E-04**	14	1.37E-03	**2.74E-03**
ADHD	411	23	3.75E-02	**4.50E-02**	11	2.03E-02	**2.91E-02**	9	4.64E-04	**1.27E-03**
Bipolar Disorder	850	45	1.29E-02	**1.94E-02**	25	1.51E-04	**4.96E-04**	13	8.37E-04	**1.79E-03**
Depressive Disorder	950	64	4.37E-06	**2.18E-05**	25	7.81E-04	**1.79E-03**	13	2.26E-03	**3.99E-03**
Schizophrenia	1965	115	9.21E-07	**1.38E-05**	50	3.88E-06	***2.18E-05***	28	2.19E-06	***2.18E-05***
Alzheimer's Disease	2061	123	1.25E-07	***3.76E-06***	36	4.64E-02	5.36E-02	22	1.89E-03	**3.54E-03**
Parkinson' Disease	409	22	5.85E-02	6.50E-02	8	1.71E-01	1.77E-01	6	2.60E-02	**3.55E-02**
Glioma	2389	115	2.89E-03	**4.82E-03**	39	8.85E-02	9.48E-02	11	7.80E-01	7.80E-01

### Identification of hub genes

**Figure 5:**
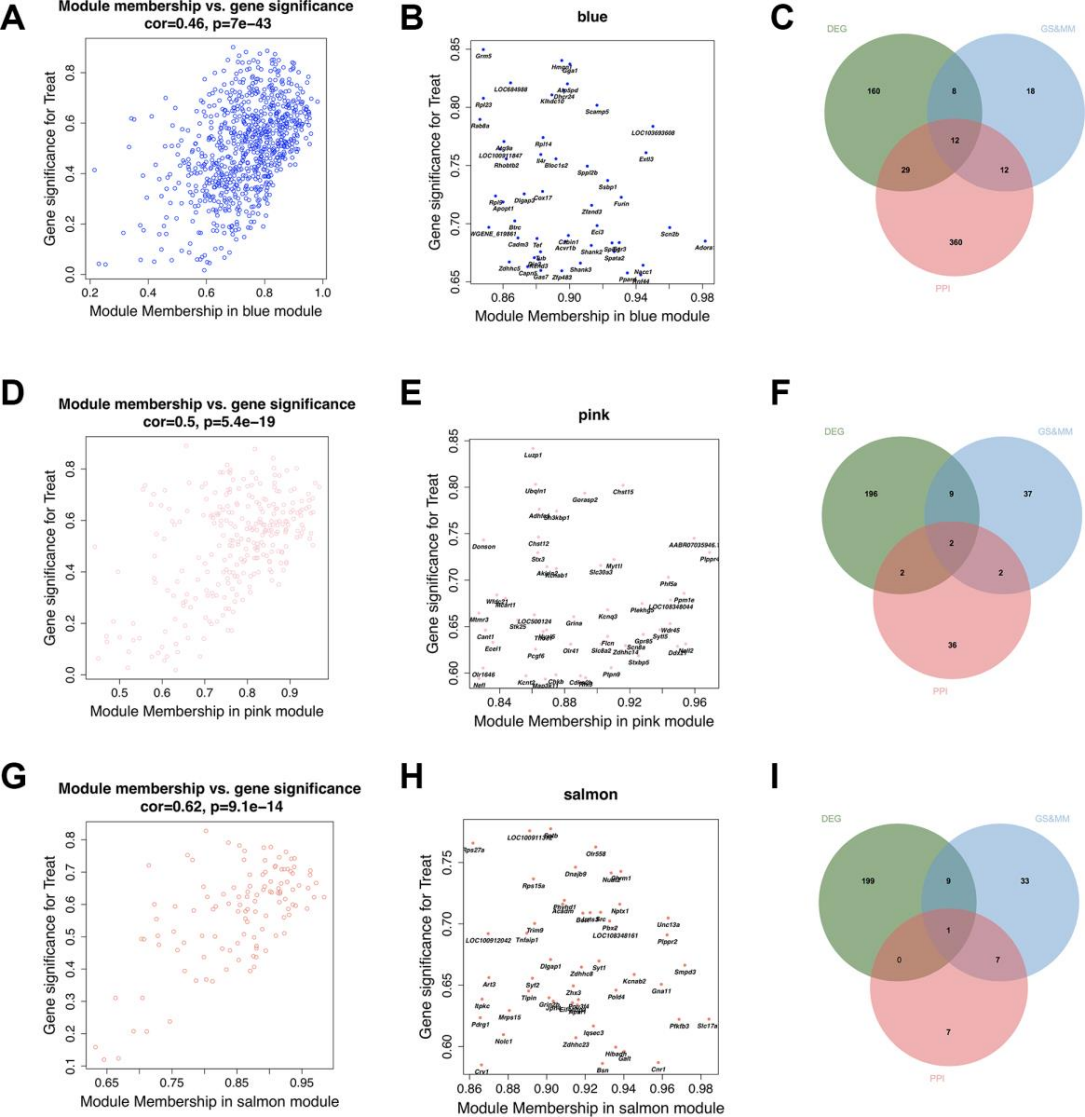
**Identification of final hub genes from the hippocampus tissue.** Scatter plotting of the correlation between gene significance (GS) and module membership (MM), top 50 module genes with high GS and MM, and Venn diagram of the overlapping genes belonging to the PPI network with degree ≥ 5, top 50 module genes with high GS and MM, and the differentially expressed genes (DEGs) in the blue (**A**–**C**), pink(**D**–**F**), and salmon (**G**–**I**) modules.

As shown in Figure 5A, 5D and 5G, the Factor-associated down-regulated blue, pink and salmon modules were significant positive correlations between GS and MM in the hippocampus. This showed that these three modules have high quality. We selected the top 50 genes in each of the three modules (Figure 5B, 5E, and 5H) with high GS and MM from blue, pink and salmon modules, (Supplementary Table 4) respectively. And we also showed the top 50 high connectivity genes based on the co-expression network, which may provide a basis for other studies (Figure 7A–7C, Supplementary Figure 6A–6C). We constructed PPI networks of these three module genes using STRING and highlighted the top 50 high connectivity genes (Figure 7D–7F, Supplementary Figure 6D–6F). Hub genes selection was based on features including significantly different between prenatal nutrition deficiency and control offspring, high GS and MM and connection degree ≥ 5 in the PPI network (Figure 5C, 5F, and 5I). We found twelve hub genes in the blue module, including *Hmgn1, Ssbp1, LOC684988, Rpl23, Gga1, Rhobtb2, Dhcr24, Atg9a, Dlgap3, Grm5, Scn2b* and *Furin*, two hub genes in the pink module, including *Sh3kbp1* and *Ubqln1*, and one hub gene (*Unc13a*) in the salmon module (Supplementary Figure 7A).

### qPCR validation of the hub genes

To verify the main conclusion drawn from the microarray results, the relative expression levels of the 15 key genes, including *Hmgn1, Ssbp1, LOC684988, Rpl23, Gga1, Rhobtb2, Dhcr24, Atg9a, Dlgap3, Grm5, Scn2b, Furin, Sh3kbp1, Ubqln1*, and *Unc13a*, were determined using qPCR. No significant difference in body weight and gender were detected between the two groups of rats. The qPCR analysis results indicated that the expression levels of *Hmgn1, Ssbp1, LOC684988, Rpl23, Gga1, Rhobtb2, Dhcr24, Dlgap3, Scn2b, Furin, Ubqln1*, and *Unc13a* were significantly up-regulated in samples from RLP50 group compared with the prenatal normal nourished group, whereas *Grm5* and *Sh3kbp1* were significantly down-regulated. Meanwhile, there were no significant differences in the expression level of *Atg9a* between the two groups.

### Inference of miRNA–mRNA-transcription factor interactions

**Figure 6:**
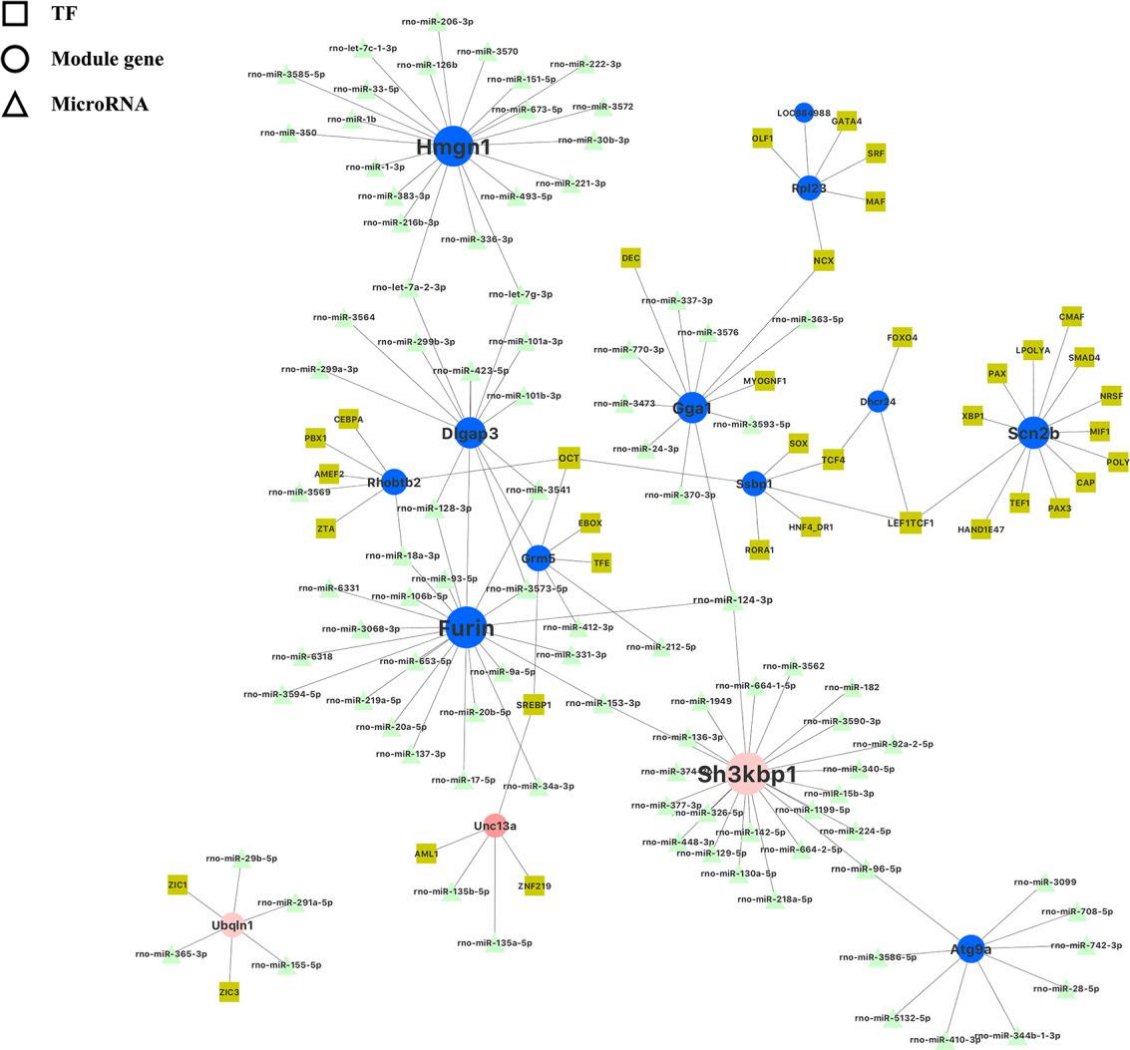
**Illustration of miRNA–mRNA-transcription factor interaction for hub genes identified in the blue, pink and salmon modules in the hippocampus.** Each hub gene is denoted as solid circle; each transcription factor is denoted as solid square; and each miRNA is denoted as a solid delta.

The final “real” 15 hub genes were simultaneously input into the miRDB database to identify the microRNAs target sites, and were combined with DiRE database to identify transcription factors regulating these hub genes (Figure 6, Supplementary Table 10). We found a transcription factor, Cebpa, was differentially expressed with statistical significance (Supplementary Figure 7B). Cebpa may transcribe the hub gene Rhobtt2 in the blue module, whereas miR-3569 and miR-18a-3p may suppress the translation of Rhobtt2. Meanwhile, miR-18a-3p may also suppress the translation of hub gene *Furin* in the blue module, which may cause the indirect correlated expression between *Rhobtt2* and *Furin* in the blue module. All hub genes are co-regulated through miRNAs, or transcription factors, or other potential novel regulators of transcription and post-transcription dominating prenatal nutritional deficiency-induced gene expression changes. For example, miR-let-7g-3p can regulate both *Hmgn1* and *Dlgap3*; meanwhile the transcription factor NXC may regulate both *Rpl23* and *Gga1* in the miRNA–mRNA-transcription factor interaction network. Detailed illustration of the inferred miRNA–mRNA-transcription factor interactions for the 15 hub genes is present in Figure 6.

**Figure 7:**
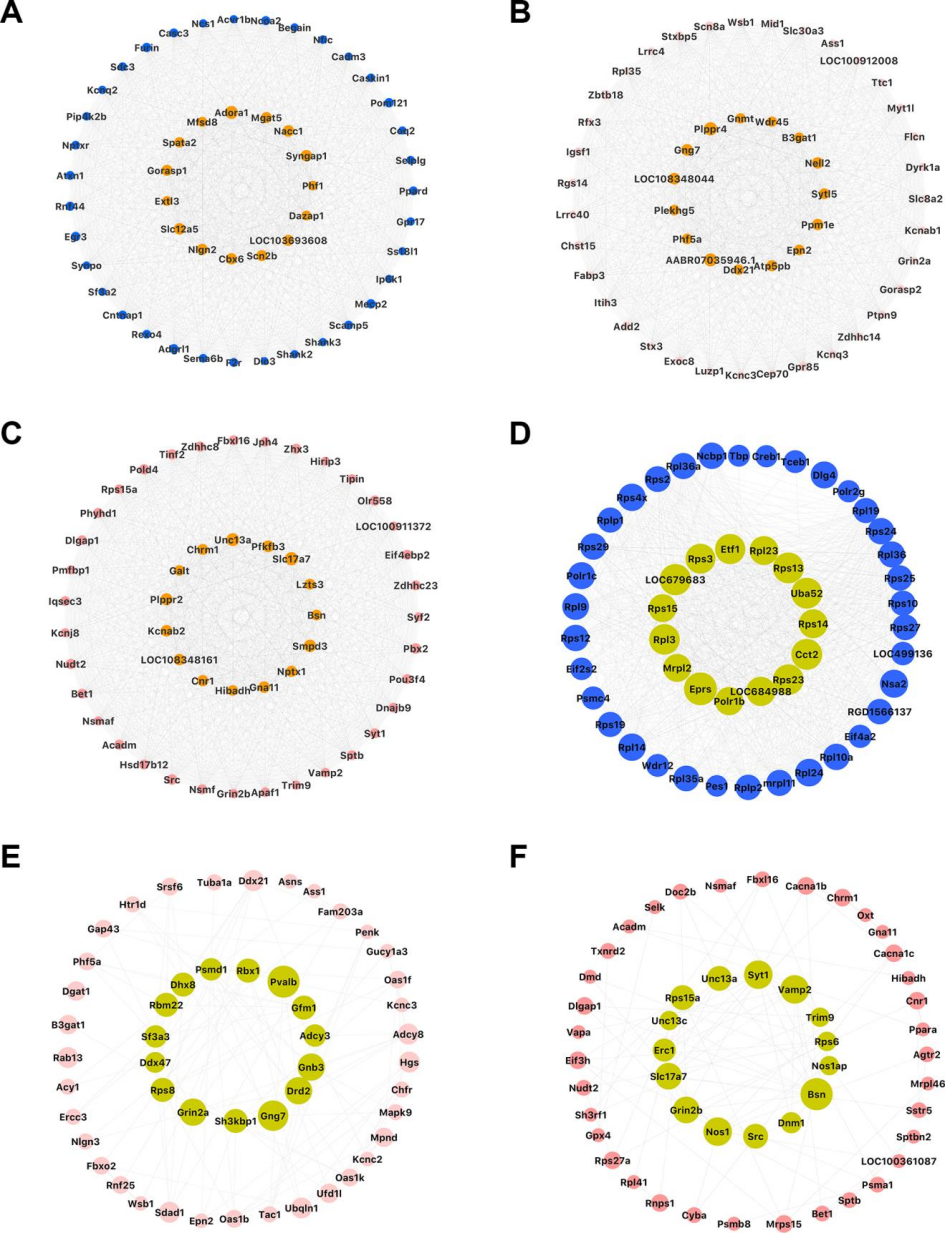
**Top 50 genes with high network connectivity.** (**A**–**C**) Top 50 genes determined through co-expression networks in the blue, pink, and salmon modules, respectively; (**D**–**F**) Top 50 genes determined through PPI networks in the blue, pink, and salmon modules, respectively.

In the study of hub genes, we additionally screened the Top50 connectivity gene (Figure 7A–7C, Supplementary Figure 6A–6C, Supplementary Tables 8 and 9) in the co-expression network and the Top50 connectivity gene (Figure 7D–7F and Supplementary Figure 6D–6F) in the PPI network. These contents are not highly related to the main ideas of this article, but could provide some basis for the follow-up study.”

## DISCUSSION

In our previous study, we demonstrated that prenatal exposure to malnutrition results in systematical changes of transcriptome and DNA methylome associated with neuropsychiatric diseases [12]. However, the changes in gene expression and modifications were generally mild, and we focused on individual DEGs and the correlation between epigenetic and transcriptome programming. In the present study, we performed weighted gene co-expression network analysis to investigate the joint effect of multiple genes and key regulators. We identified three genetic regulatory subnetworks, fifteen key regulating genes along with their regulating transcription factors and microRNAs in rat hippocampus perturbed by a prenatal nutrition deficiency. These findings provide a further understanding of how prenatal nutritional deficiency perturbs hippocampus development through transcriptomic regulatory network level. We did not identify gender -specific changes in gene co-expression networks in the hippocampus and the prefrontal cortex. We thought this was plausible since prenatal exposure to famine increases the risk of schizophrenia in both males and females [1, 2]. Our rat model (RLP50 offspring) simulated the prenatal malnutrition occurred in famine. Compared with the widely used prenatal protein malnutrition model, in which a low protein diet would be used five weeks before mating [14–16], our treatment was more severe but limited to the gestational period. The difference between the two models may result in different findings associated with prenatal malnutrition. The findings from our RLP50 model reflected the role of prenatal extreme malnutrition on the brain development of embryo and fetus, while those findings in the prenatal protein malnutrition model might probably be caused by abnormal development of oocytes as well.

Consistent with previous findings, prenatal protein malnutrition has a more severe impact on the hippocampus than in the prefrontal cortex [17–19]. For example, studies have shown that protein malnutrition during pregnancy reduced the total number of neurons in the hippocampus CA1 subarea and had important implications for the perisomatic of hippocampal pyramidal neurons, which will have an important impact on the formation of the hippocampus [5]. Based on the results of network analysis, we conducted a preservation analysis of the expression profiles between the hippocampus and prefrontal cortex (Supplementary Figure 5). The results showed that there were some modules in the hippocampus that were not preserved with those in the prefrontal cortex. These findings may indicate that there are regional differences in the effects of maternal nutritional deficiency on the genetic changes of the offspring brain. The hippocampus plays an important role in learning and memory and is believed to be crucially involved in the pathophysiology of many neuropsychiatric disorders, including schizophrenia and autism. And many reports have found that hippocampus dysfunction is caused by deficits of some gene expression in synapses in subjects with schizophrenia, or autism, and that the changes probably result from altered brain development rather than tissue damage [20]. The most commonly reported genes associated with schizophrenia and autism were expressed during prenatal life and affected prenatal development in multiple brain regions, including the hippocampus [21, 22]. Therefore, the impairment of hippocampal development and functions may be important for the increased risk of schizophrenia and autism after prenatal exposure to malnutrition. The prefrontal cortex is responsible for complex cognitive behavior and its dysfunction is associated with many neuropsychiatric disorders. However, we did not identify the genetic regulatory subnetworks and key regulating genes in the prefrontal cortex perturbed by prenatal nutrition deficiency. A possible explanation is the statistic corrections for multiple tests. In this study, the differentially expressed genes were screened after rigorous correction, and therefore a much smaller number of DEGs was identified compared with our previous report, which used real-time PCR to validate DEGs [12]. Neuropsychiatric disorders are multifaceted diseases with complex and heterogeneous etiology. Prenatal nutrition deficiency alone may have a limited impact on prefrontal cortex development. In this study, malnutrition was restricted in the gestational period. It has been known that the stress during gametal or postnatal development affects brain functions and gene expression [23, 24]. Moreover, neuropsychiatric disorders have strong but largely undiscovered heritable components [25]. Prenatal nutrition deficiency may exert their effect on the prefrontal cortex in a specific genetic background.

Network analysis screened out three modules associated with prenatal malnutrition in the hippocampus, including blue, pink and salmon one (Figure 2A). The GO and KEGG analysis suggested genes in these modules were involved in synaptic development, neuronal projection, cognitive function, and learning function. Cell-types analysis showed that salmon module significantly enriched hippocampal CA1 pyramidal neurons (Figure 4, Supplementary Table 7). The abundance of pyramidal neurons in the hippocampus suggests that their proper function is necessary for cognitive processing, and thus defects in pyramidal neuron function could be a reason for the cognitive impairment in neuropsychiatric disorders, such as Alzheimer’s disease or schizophrenia [26]. Overlap analysis of genes associated with major brain diseases demonstrated that dysregulated genes associated with Alzheimer's disease, autism, and schizophrenia were overrepresented. All these findings based on enrichment analysis demonstrated that these three modules in rat hippocampus represented the key gene regulatory networks and pathways related to the impact of prenatal malnutrition on neuropsychiatric diseases, including Alzheimer's disease, autism, and schizophrenia (Table 2).

Screening of hub genes is based on three conditions, including: whether it is a differentially expressed gene; whether it is a high GS and high MM gene in the co-expression network: and whether the connectivity in the PPI network is more than 5 (Figure 5). The finally identified15 hub genes, which were differentially expressed in PLP50 offspring, showed high GS and high MM scores, and have more than 5 connection degrees in PPI network, were compelling candidates for the key regulators responding to the prenatal nutritional deficiency.

Twelve hub genes were identified in the blue module, including *Hmgn1, Ssbp1, LOC684988, Rpl23, Gga1, Rhobtb2, Dhcr24, Atg9a, Dlgap3, Grm5, Scn2b*, and *Furin*. The hub gene *Hmgn1* encodes the high mobility group N1 protein that affects the structure and function of chromatin [27]. It locates on chromosome 21 and over-expressed in Down syndrome. It has been reported that Hmgn1 regulated the expression of methyl CpG binding protein 2 (MeCP2) by altering the chromatin structure and histone modification of its promoter. Changes in the expression level of *Hmgn1* can lead to abnormal activity and anxiety in mice. MeCP2, a well-know gene for Rett syndrome, was also differentially expressed in our original microarray data. These results suggested that Hmgn1 affected the psychiatric behavior of mice and these epigenetic changes caused by changes in its expression level may play a role in neurodevelopmental disorders, such as Rett syndrome and autism [28]. Rhobtb2, an atypical Rho GTPases, plays an important role in synaptic plasticity and cognitive function. Missense variants in *Rhobtb2* have been reported to cause a developmental and epileptic encephalopathy in humans, and altered levels triggering neurological defects in drosophila [29]. To further understand the role of these hub genes, we employed an online database to model the interaction of the upstream regulating factors including transcription factors and microRNAs with these hub genes (Supplementary Table 5). And we further identified Cebpa, a transcription regulatory factor of *Rhobtb2*, was differentially expressed after exposure to prenatal malnutrition (Supplementary Figure 7B). Cebpa may promote the transcription of the hub gene Rhobtt2 in the blue module, whereas miR-3569 and miR-18a-3p may suppress the translation of Rhobtt2. Meanwhile, miR-18a-3p may also suppress the translation of hub gene *Furin* in the blue module, which may cause the indirect correlated expression between *Rhobtt2* and *Furin* in the blue module. One study found that schizophrenia-associated rs4702 G allele-specific down-regulation of *Furin* by miR-338-3p reduces mature BDNF production, which affected neurodevelopmental disorder [30]. GRM5 (coding for metabotropic glutamate receptor 5, mGluR5) is a promising target for the treatment of cognitive deficits in schizophrenia, which may be related to the reduction of hippocampal volume [31]. People have reported that Dlgap3 and its interaction protein Slc1a1 may serve drug targets of some neuropsychiatric disorders, which are associated with adverse reactions to atypical antipsychotics (AAP), including obsessive-compulsive disorder in schizophrenia [32, 33]

Two hub genes in the pink module were identified, including *Sh3kbp1* and *Ubqln1*. *Ubqln1* is highly correlated with differentially expressed genes associated with neurodegenerative diseases, like Alzheimer’s disease, and may affect neurodevelopment [34] (Supplementary Figure 7A).

The salmon module enriched pyramidal neurons (Figure 4, Supplementary Table 7) in the hippocampal CA1 region. One hub gene named *Unc13a* was identified in this module. The hub gene *Unc13a* encodes a protein bind to phorbol esters and diacylglycerol and play important roles in neurotransmitter release at synapses. A new heterozygous mutation of *Unc13a* was reported in a patient with dyskinesia, cognitive retardation, speech disorder, autism and hyperactivity [35]. Findings from multiple GWAS analyses demonstrated that *Unc13a* is associated with Alzheimer’s disease [36, 37].

There were some limitations in our study. In our RLP50 model, we mainly focused on maternal protein deficiency that would result in low birth weights. However, some micronutrients, such as folate, Vitamin D, and iron, may also be involved in the association between famine and neurodevelopment and related neuropsychiatric disorders. It will be of great interest to compare our results to those where the reprogramming of gene expression and epigenetic modifications occur in models in which only specific micronutrients are restricted. Meanwhile, we inferred an undirected network in which connectivity between nodes does not indicate the causal regulatory relationships.

In conclusion, these findings demonstrated that these genes with the three modules in rat hippocampus involved in synaptic development, neuronal projection, cognitive function, and learning function are significantly enriched hippocampal CA1 pyramidal neurons and suggest that three genetic regulatory subnetworks and thirteen key regulating genes in rat hippocampus perturbed by a prenatal nutrition deficiency. These hub genes and their related miRNA–mRNA-transcription factor interaction subnetworks, which dominate prenatal nutritional deficiency-induced gene expression changes, may be prenatally involved in the etiologies of some neuropsychiatric disorders, including Alzheimer’s disease, autism, and schizophrenia. Further *in vivo* and *in vitro* functional analyses warrant deciphering the precision biological mechanisms on how the potential novel regulators of transcription and post-transcription, including micro-RNAs, long noncoding RNAs, and transcription factors, regulate the transcription-level RNA interactions.

## MATERIALS AND METHODS

### Animal model

Sprague-Dawley rats (Shanghai Slack Laboratory Animal Co. Ltd.), were used in all experiments under the Guide for the Care and Use of Laboratory Animals (Washington, DC: National Academic Press). All experimental procedures and protocols were approved by the Institutional Animal Care and Use Committee at the Shanghai Jiao Tong University.

Seventeen Sprague–Dawley female rats were randomly separated into three groups on the first day after mating, and were fed their respective diets until E18 (embryo, 18 days), two days before parturition (control: 5 dams; malnourished group [LP]: 5 dams; famine group [RLP50]: 7 dams). The control group was given a standard rodent diet (20% protein, Research Diets, Inc. D12450B) and water *ad libitum* and the LP group was given a low-protein (6% protein, Research Diets, Inc. D06022301) diet and water *ad libitum*. The RLP50 group was given 50% of the LP group’s low-protein diet, reflecting both the protein malnutrition and food-deficiency likely to prevail during the famine. All the female rats were fed with the standard rodent diet after E18. After parturition, all litters were culled to 8 pups, which were fostered by their own mothers. On day 21, all pups were weaned and placed on a standard rodent diet. Rats were raised to be 10 weeks old and were anesthetized before being killed. The PFC and hippocampus from the adult offspring of both control (n = 60) and RLP50 (n = 60) rats were then isolated and stored in RNAlater® Solution Life technologies). The treatment of the pregnant rats in RLP50 was according to our previous model [12, 13]

### Microarray data pre-processing and identification of DEGs in the hippocampus and prefrontal cortex

Total RNA was extracted from the hippocampus and prefrontal cortex tissues derived from 10 control and 10 RLP50 rats using mirVana™ miRNA Isolation Kit. The RNA integrity number (RIN) was detected by Agilent Bioanalyzer and the samples with RIN > 7.5 were selected for microarray gene expression analysis using NimbleGen Rat Gene Expression 12 x 135K Arrays. (Supplementary Table 1) Quantile normalization of microarray data was performed to obtain standardized using preprocessCore package in R [38] (Supplementary Figure 1A, 1B, 1H, 1G and Supplementary Table 2). A total of 26419 microarray probes were converted into 9532 Ensembl gene IDs by using the biomaRt package [39]. Samples defined as outliers based on standardized sample network connectivity Z-scores < -2 were removed [40] (Supplementary Figure 1C and 1I). The batch effect of expression profiles was treated by using the ComBat function of the sva package in R (Supplementary Figure 1D, 1E, 1J, 1K, and Supplementary Table 2). We balanced maternal prenatal nutrition deficiency/control status across gender and there was no significant difference in gender between the two groups (p-value > 0.05) (Supplementary Figure 1F and 1I). Differential expression analysis using a linear empirical Bayes model based on the limma package [41]. Differentially expressed genes with statistical significance were identified after p-value correction with a false discovery rate (FDR).

### Weighted gene co-expression network construction

To assess the inter-correlation of the intensities of the 9532 Ensembl gene IDs (that remained after preprocessing) and the relationship between them and feature data, signed co-expression analysis was performed using the WGCNA R package. The pair-wise correlation matrix was calculated by biweight midcorrelation, and an adjacency matrix is calculated by raising the correlation matrix to power [42]. Based on a fit to scale-free topology (R2 > 0.8) [40] (Supplementary Figure 4D and 4H), the powers of 15 (Supplementary Figure 4A and 4B) and 18 (Supplementary Figure 4E and 4F) were used for the construction of hippocampus and prefrontal cortex networks, respectively. Next, the dynamic tree cut methods were used to construct the basic network and the topological overlap dissimilarity matrix (1-TOM) was then used as input average linkage hierarchical clustering to create the network dendrogram [43] (Supplementary Figure 4C and 4G). For the basic network, we set the minimum number of modules to be 100 and the deep split parameter in 2 for a medium sensitivity of cluster splitting. Finally, modules were defined as branches of the dendrogram, which was cut based on the hybrid dynamic tree-cutting method [40, 42, 43].

Modules were summarized by their first principal component (ME, module eigengene) and the mergeCloseModules function in the WGCNA R package was used to merged modules whose eigengenes were more than 0.85 correlated [40]. We define module membership of each gene in each module (kME) by correlating its gene expression values with the module eigengene of a given module [44]. Pearson correlation values between MEs and “Factor” (famine offspring vs control offspring) or “Gender” were calculated, and p-values were adjusted with FDR. For each module, the correlation of two parameters [the gene significance (GS) and the module membership (MM)] was evaluated. GS stood for the magnitude of the correlation between the intensities of individual expression genes in the module and “Factor”, and MM meant the magnitude of the correlation between the intensities of individual expression genes in the module and MEs of the module.

### Module preservation test between the hippocampus and prefrontal cortex

To examine whether the co-expression structure (density and connectivity) is similar between the hippocampus and prefrontal cortex samples, we conducted module preservation analysis based on the modulePreservation function in the WGCNA R package. We used a composite preservation statistics method to analyze the conservativeness of the module [45]. For each module, the Z-summary statistic was used to measure module density and intramodular connectivity metrics. In correlation networks, the 4 density preservation statistics are summarized by Zdensity (function 1), the 3 connectivity based statistics were summarized by Zconnectivity (function 2), and for each module, the Zsummary (function 3) was used to measure that combines module density and intramodular connectivity metrics defined as follows:

Z_density_ = median (Z_meanCor_, Z_meanAdj_, Z_propVarExpl_, Z_meanKME_) (function 1)

Z_connectivity_ = median (Z_cor.kIM_, Z_cor.kME_, Z_cor.cor_) (function 2)

Z_summary_ = (Z_density_ + Z_connectivity_)/2 (function 3)

We usually defined: if Z_summary_ > 10 indicated high preservation among modules; if 2 < Z_summary_ < 10 indicated weak to moderate preservation; if Z_summary_ < 2 indicated no evidence that the module preserved.

The module size has a great influence on Z statistics. Therefore, when comparing the preservation statistics of different sized modules, we need to conduct medianRank for preservation analysis. It shows that modules with lower median rank tend to show stronger preservation statistics than those with higher median rank.

### Functional enrichment analysis of modules associated with prenatal nutritional status

Gene ontology (GO) terms, including biological processes, molecular functions and cellular components, and KEGG pathway enrichment analyses in the modules, were conducted with the R package, clusterProfiler (version 3.5) [45]. We used the annotation function in clusterProfiler to group significant GO terms and pathways based on co-associated genes to remove redundant terms. GO terms and pathway groups were considered significant when the FDR-corrected P-value of the clustered GO terms and pathways is below 0.05.

Cell-type enrichment analysis for interesting modules was conducted using the R package anRichment [44]. For enrichment analysis of cell-type-specific genes, we used cell type-specific genes identified in gene expression datasets from neurons, astrocytes, myelinating oligodendrocytes, microglia, and endothelial cells [46]. Genes were considered cell-type specific, if their expression levels had more than ten-fold higher compared to the mean levels of expression in the other cell types. The statistical significance of enrichment was determined by Fisher’s exact test.

The R package GeneOverlap(version 1.22.0) [47] was employed for overlap analysis of the module genes with disease-related genes annotated in a database named DisGeNET (http://www.disgenet.org) to evaluate if the modules are enriched with neuropsychiatric genes. This package uses Fisher's exact test to calculate the significance of each pair of gene lists in comparison to the genomic background. The function returns the number of intersecting genes between the two lists, the P-value, and the estimated odds ratio. The null hypothesis is that the odds ratio is no larger than 1, and values larger than this indicate a positive association between two lists. Therefore, if a disease gene set in DisGeNET is significantly overlapped with the gene set of a module responding to prenatal nutritional deficiency, it indicates that this disease is closely related to hippocampus dysregulation responding to prenatal malnutrition. All analyses with an FDR adjusted P-value of < 0.05 were selected as statistical significance.

### Protein-protein interaction network construction and identification of hub genes in interesting modules

The Protein-Protein Interaction (PPI) network of the genes in the selected co-expression module was constructed according to the STRING v11.0 database [48] and was visualized using Cytoscape.

Hub genes have high connectivity within a gene module and are functionally significant [44]. In our study, after an interesting module was chosen, hub genes were defined by top50 module genes with high gene significance (GS) and module membership (MM). In the PPI network, genes with a combined score of ≥0.6 and a connectivity degree of ≥5 were also defined as hub genes. The shared hub genes in both the co-expression network and PPI network were regarded as “real” hub genes for further analyses. A Venn diagram (http://bioinformatics.psb.ugent.be/webtools/Venn/) was constructed to identify these “real” hub genes [49].

### Verification of hub genes with qPCR based on independent samples

Hub genes were selected for validation using quantitative real-time polymerase chain reaction (qPCR) in the different samples including 40 prenatal malnourished rats(RLP50 group) and 40 prenatal normal nourished rats(control group), as used for the microarray experiments. For the quantification of mRNAs, 1 μg of the total RNA was converted into cDNA using a reverse transcription kit (Promega) following the manufacturer's instruction. The TaqMan® Gene Expression Assay was then used to detect the gene expression of the hub genes. The qPCR was conducted using SYBR Premix Ex Taq (Takara, Japan) in 20 μl reaction solution containing 10 μl SYBR Premix Ex Taq (2X), 0.4 μl forward primer and 0.4 μl reverse primer, 0.4 μl ROX reference dye, 1μl cDNA and 8.2 μl ddH2O. The PCR amplification procedure was carried out at 95°C for 10 s; 40 cycles of 95°C for 5 s and 60°C for 34 s; followed by disassociation curve analysis in an ABI 7500 fast real-time PCR system (Applied Biosystems, USA). The amplification reaction without the template was used as a no template control. All reactions were performed in triplicate. The β-actin gene was used as an internal reference. Primers used for gene amplification are available on request. The relative mRNA abundance was calculated using the 2-ΔΔCt method. Statistical comparison of the levels was analyzed using two-tail unpaired Student's t-test, and differences were considered significant if P < 0.05.

### Modeling miRNA–mRNA-transcription factor interactions for hub genes

To explore transcription factors (TFs) and miRNA–mRNA interaction in particular modules and to predict the possible regulatory relationships between them that dominate prenatal nutritional deficiency-induced gene expression changes, we used two common online databases, respectively. The TFs that bind onto hub genes were screened using DiRE [50] (https://dire.dcode.org), a web server can predict common REs for any set of input genes. microRNAs of hub genes were screened using miRDB [51] (http://mirdb.org/mining.html). This database collected miRNA-genes regulatory relationships.

## Supplementary Materials

Supplementary Figures

Supplementary Table 1

Supplementary Table 2

Supplementary Table 3

Supplementary Table 4

Supplementary Table 5

Supplementary Table 6

Supplementary Table 7

Supplementary Table 8

Supplementary Table 9

Supplementary Table 10
