# Supplementary material for: Genetic regulatory subnetworks and key regulating genes in rat hippocampus perturbed by prenatal malnutrition: implications for major brain disorders
Source: Aging (Albany NY). 2020 May 11;12(9):8434–58. doi: 10.18632/aging.103150 (PMC7244046; doi:10.18632/aging.103150)
Supplement: Supplementary Figures [file aging-12-103150-s007.pdf]

SUPPLEMENTARY FIGURES

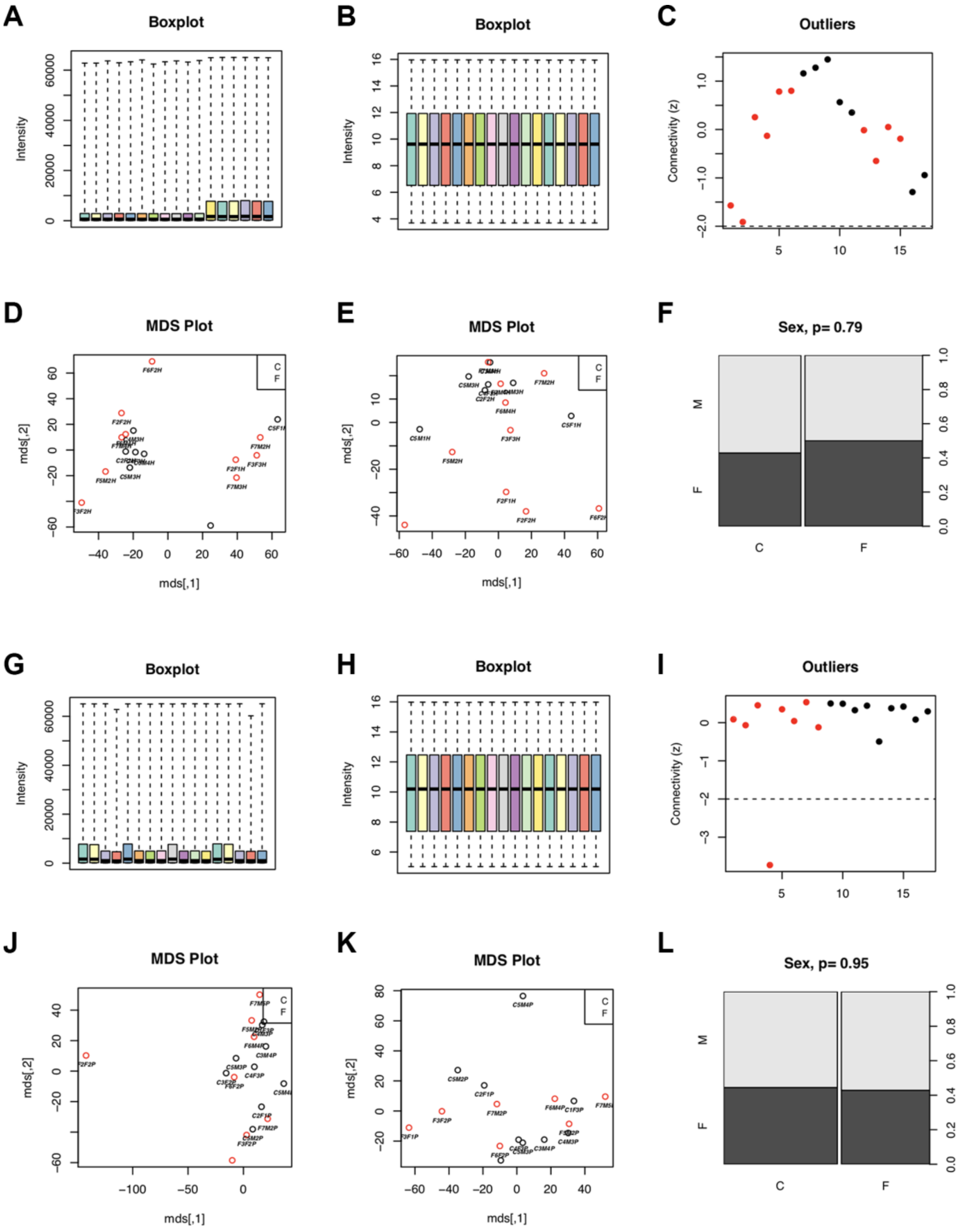

Supplementary Figure 1. Quality control and data preprocessing. (**A**, **B**) Pre- and post-normalization of expression profiles in hippocampus; (**C**) Detection of outlier based on standardized network connectivity z-scores in hippocampus; (**D**, **E**) Pre- and post-processing of batch effect in hippocampus; (**F**) Gender distribution for hippocampus samples between two groups treated with different prenatal nutritional status; (**G**, **H**) Pre- and post-normalization of expression profiles in prefrontal cortex. (**I**) Detection of outlier based on standardized network connectivity z-scores in prefrontal cortex. (**J**, **K**) Pre- and post-processing of batch effect in prefrontal cortex. (**L**) Gender distribution for prefrontal cortex samples between two groups treated with different prenatal nutritional status.

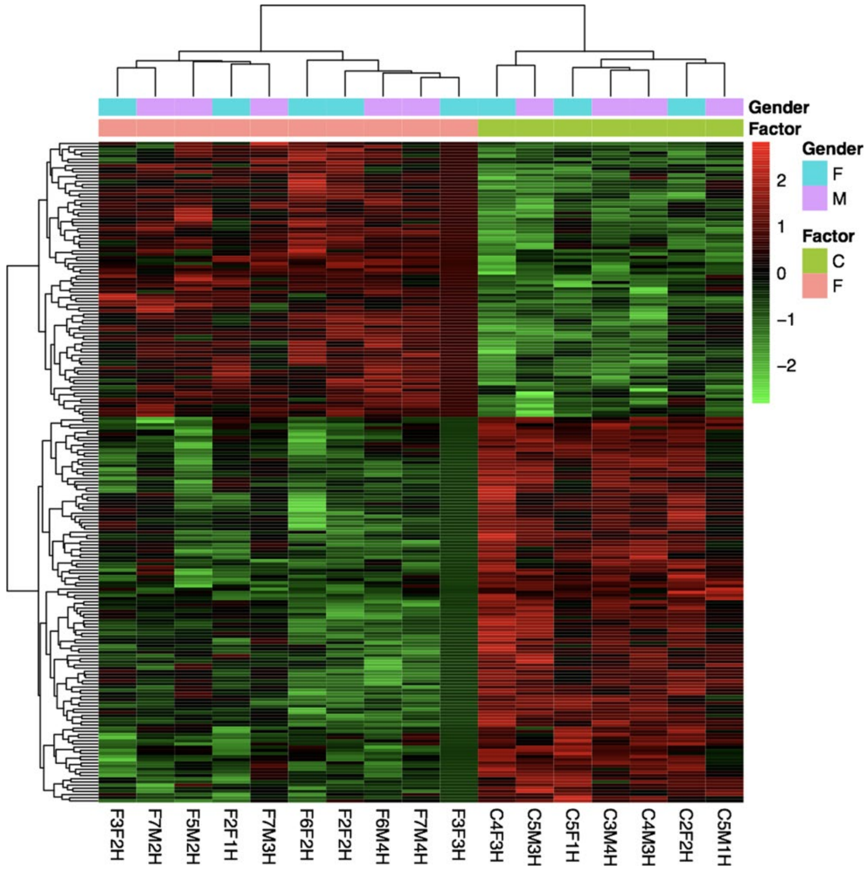

Supplementary Figure 2. Heatmap of 209 differentially expressed gene in hippocampus (Factor: C, control offspring; F, famine offspring, Gender: M, Male; F, Female).

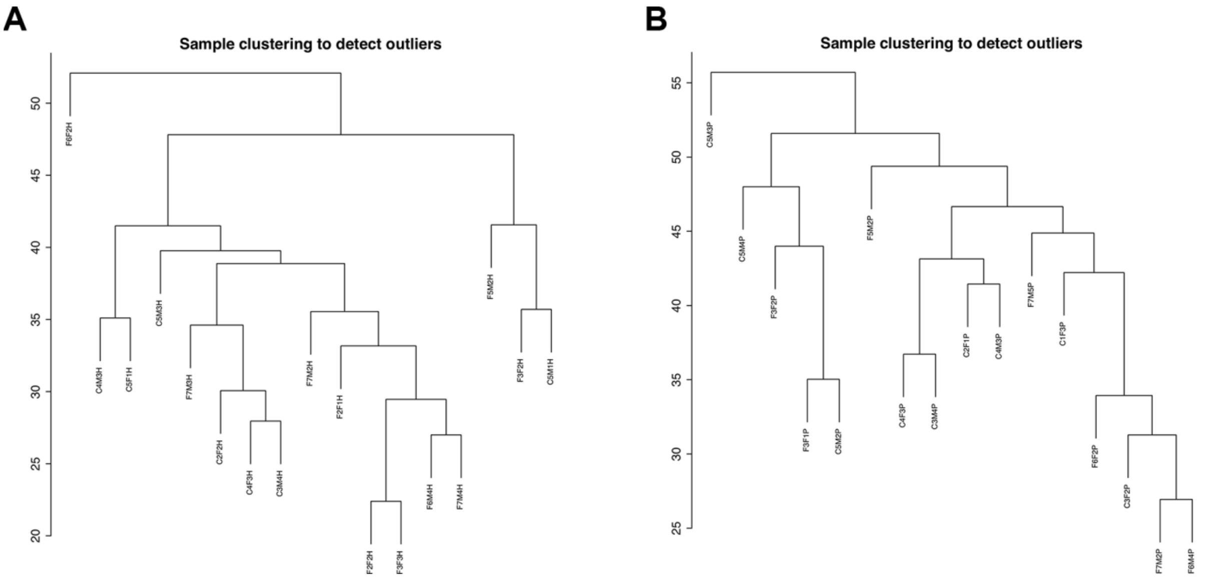

Supplementary Figure 3. Sample clustering analysis for detecting outliers in the hippocampus (**A**) and prefrontal cortex (**B**).

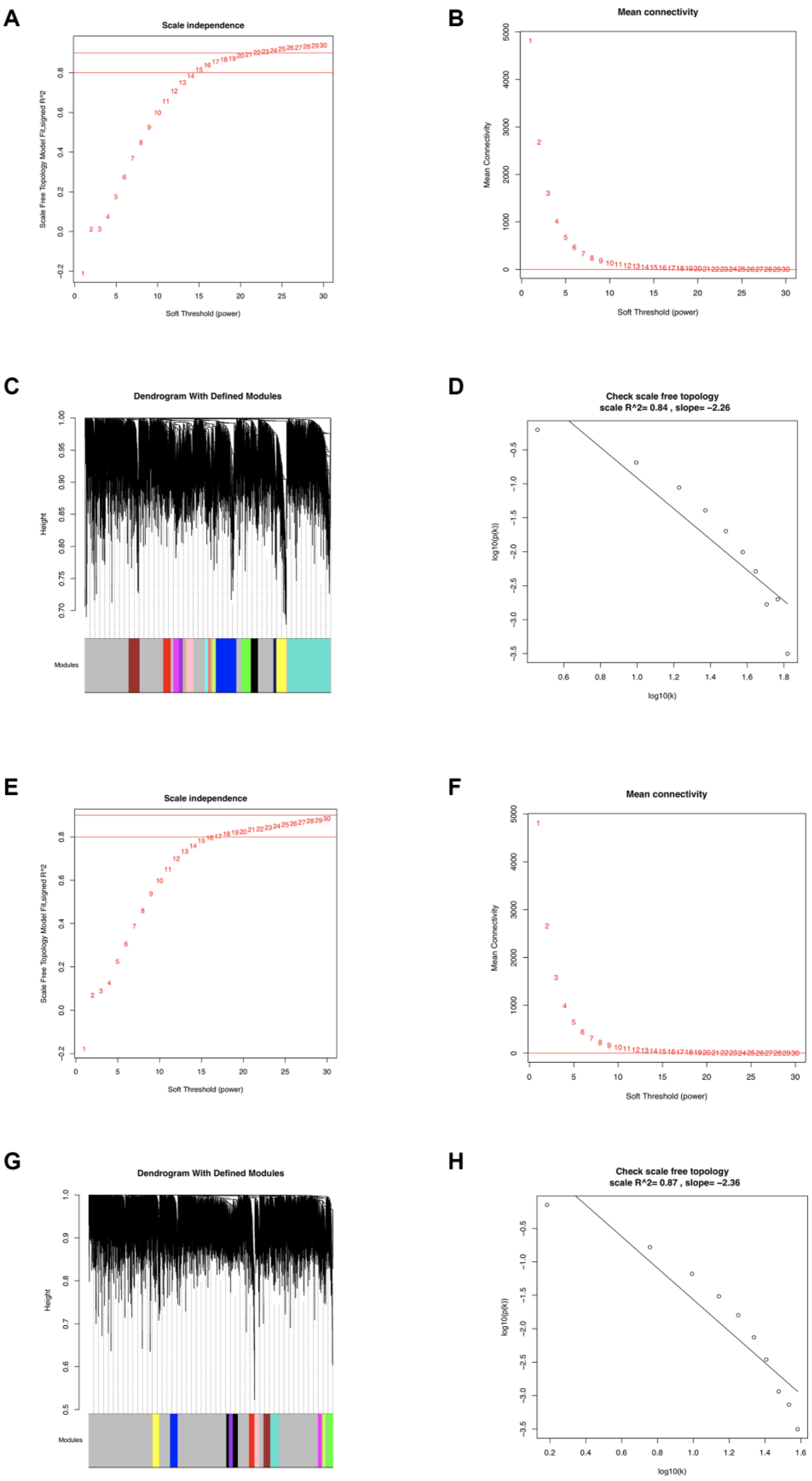

Supplementary Figure 4. Parameter selection for construction of weighted gene co-expression network in hippocampus and prefrontal cortex. (**A**) Selection of the scale-free fit index based on different soft-threshold powers (β) in hippocampus (β 15 was determined); (**B**) Selection of the mean connectivity of different soft-threshold powers in hippocampus; (**C**) Dendrogram of 9553 expressed genes clustered based on a dissimilarity measure (1-TOM) in hippocampus; (**D**) Check of scale-free topology with soft-threshold power β equaling 15 in hippocampus; (**E**) Selection of the scale-free fit index based on different soft-threshold powers (β) in prefrontal cortex (β 17 was determined); (**F**) Selection of the mean connectivity of different soft-threshold powers in prefrontal cortex; (**G**) Dendrogram of 9553 expressed genes clustered based on a dissimilarity measure (1-TOM) in prefrontal cortex; (**H**) Check of scale-free topology with soft-threshold power β equaling 17 in prefrontal cortex.

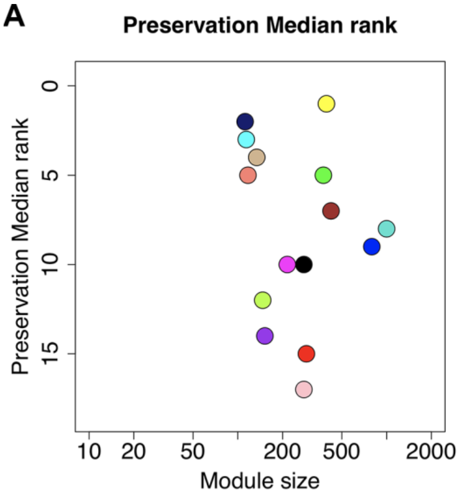

Supplementary Figure 5. Module preservation analysis between hippocampus and prefrontal cortex. The Y-axis represents preserved values and the X-axis represents module size. (**A**) median Rank test; and (**B**) Z summary statistics test.

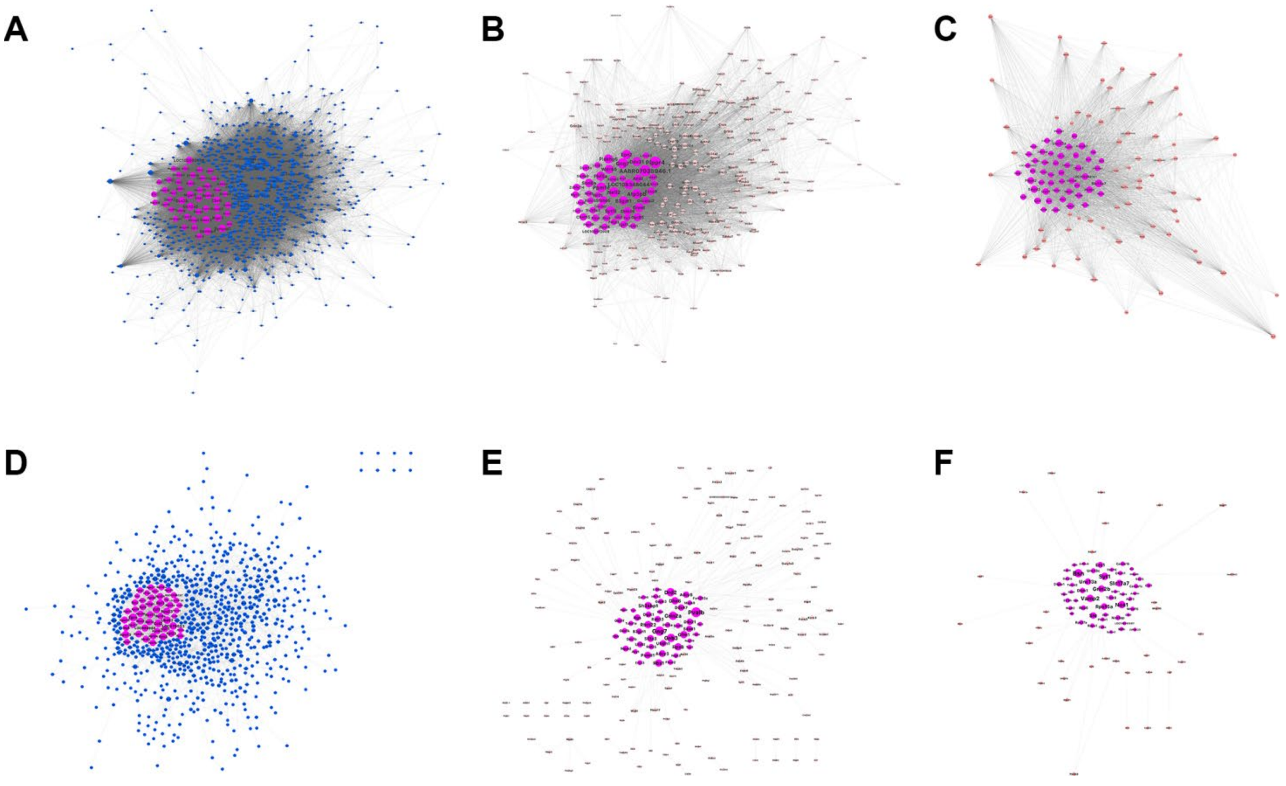

Supplementary Figure 6. Co-expression (**A**–**C**) and PPI networks (**D**–**F**) for the corresponding blue, pink, and salmon modules.

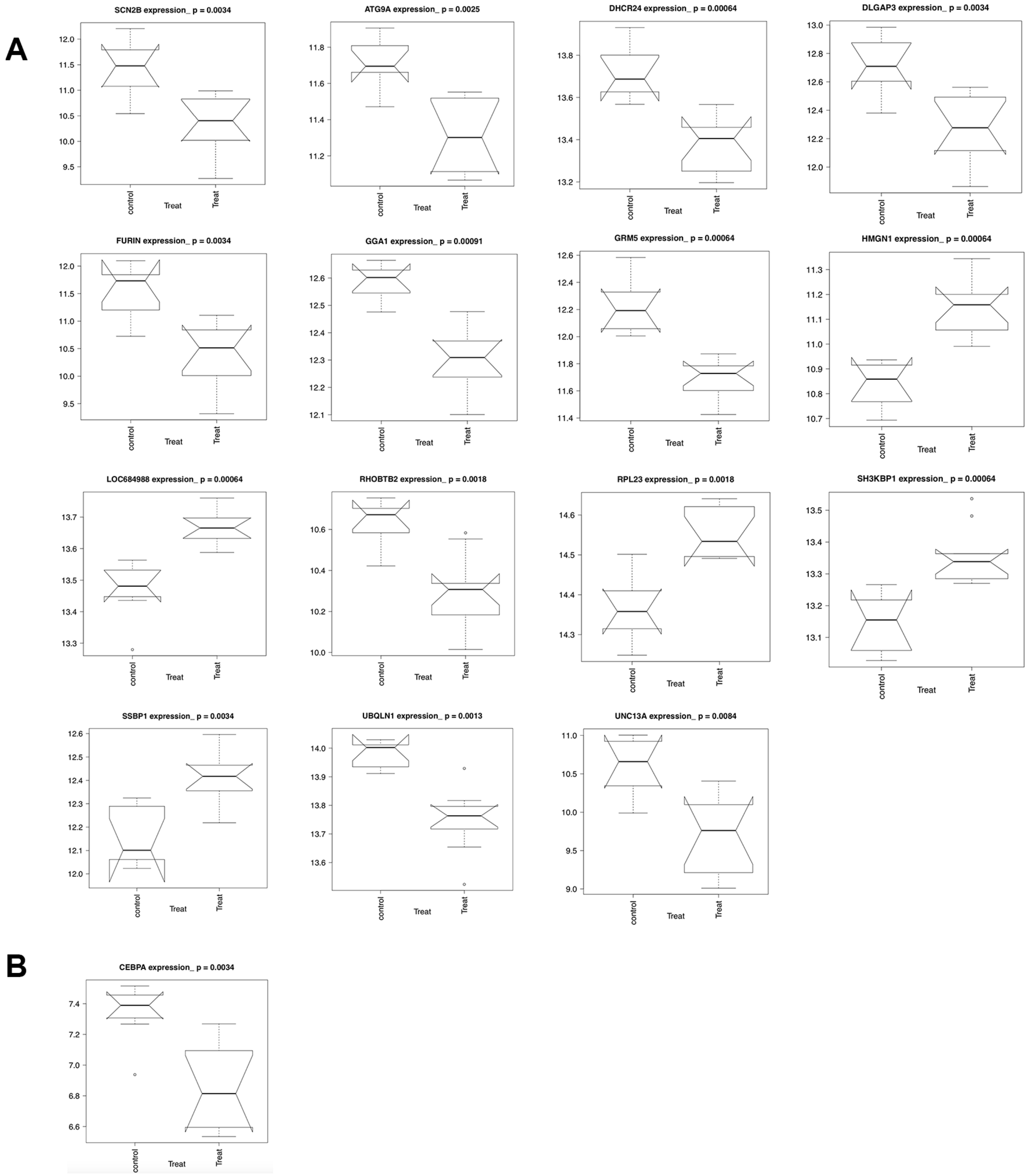

Supplementary Figure 7. Differentially expressed hub genes (**A**), and transcription factors (**B**) between two groups related to prenatal nutritional status in the interesting modules.
